# The effect of platelet-rich fibrin on normal dermal fibroblast proliferation after mitomycin-c treatment: An in vitro study

**DOI:** 10.1016/j.amsu.2021.01.093

**Published:** 2021-02-01

**Authors:** Ishandono Dachlan, Hendy Satrya Kurniawan, Aditya Wicaksana, Aditya Rifqi Fauzi, Firdian Makrufardi, Rosadi Seswandhana

**Affiliations:** Plastic Reconstructive, and Aesthetic Surgery Division, Department of Surgery, Faculty of Medicine, Public Health and Nursing, Universitas Gadjah Mada/Dr. Sardjito Hospital, Yogyakarta, 55281, Indonesia

**Keywords:** *Fibroblast proliferation*, *Mitomycin-C*, *Platelet-rich fibrin*

## Abstract

**Background:**

Disturbance in the wound healing can cause the wound turn into a chronic wound, which histologically shows fibroblast senescence with weak proliferation ability. Mitomycin-C could block cell proliferation that causes cell senescence which is similar to the chronic wound morphology. Platelet-Rich Fibrin (PRF) contains a large number of platelets, leukocytes, cytokines and growth factors. This study aims to determine whether PRF could improve the fibroblast proliferation after treatment with Mitomycin-C.

**Methods:**

Cultured normal human skin fibroblasts forth passage divided into five groups. The first group was treated with culture medium, and the second group with 10 μg/mL mitomycin-C for 2 h. The third, 4th and 5th group were treated with mitomycin-C for the same dose and period, then adding it with 100%, 50%, and 25% of PRF. The fibroblast proliferation was measured by MTT assay.

**Results:**

The fibroblast proliferation in the group with culture medium is 11.366,56 ± 4.073,32, meanwhile in the group with mitomycin-C treatment is 5.690,41 ± 2.834,22. The fibroblast proliferation in group with 100% PRF is 7.909,8 ± 3.392,19; group with 50% PRF 15.347,91 ± 8.413,02; and group with 25% PRF 13.449,56 ± 7.523,83. All of the PRF groups increased significantly compared to the group with Mitomycin-C treatment.

**Conclusions:**

Platelet-Rich Fibrin can improve normal dermal fibroblast proliferation after treatment with mitomycin-C in vitro.

## Highlights

• Mitomycin-C exposure is similar to the chronic wound morphology.
• PRF contains a large number of platelets, leukocytes, cytokines and growth factors.
• PRF groups increased significantly compared to the group with Mitomycin-C treatment.
• PRF improve normal dermal fibroblast proliferation after mitomycin-C in vitro.

## 1 Introduction

Wound healing is a very complex process because it involves various mechanisms, cells, and chemical mediators such as cytokines and growth factors. In the early stages of wound healing, platelets play a pivotal role in the release of growth factors [1]. Fibroblast holds an important role in the wound healing process, and this cell is responsible for collagen and elastin synthesis, also the extracellular matrix component organization [2,3].

The wound healing process can be delayed because of several factors. The presence of acute wound healing disruption will turn it into a chronic wound. Histologically, a chronic wound shows an image of fibroblast senescence with low proliferation ability [4].

Mitomycin-C is one of widely used chemotherapeutic drug in cancer treatment. In vitro testing of Mitomycin-C showed the presence of cell proliferation delay, both for tumors and healthy cells, and causes cell senescence similar to the morphology of a chronic wound. In vitro study by Nieto [5] with human fibroblast showed 10 μg/ml (0,03 M) Mitomycin-C for 2 h can delay the proliferation and induce apoptosis. In another in vitro study by Chen [6] revealed Mitomycin-C 0,4 mg/mL for 4 min on fibroblast can lower the normal dermis fibroblast proliferation.

Platelet-rich Fibrin (PRF) is a new platelet concentrate product made with peripheral blood centrifugation. PRF is easier and simpler in production compared to the previous platelet concentrate products. This PRF contains many cytokines and healing factors that can induce the wound healing process [6,7].

This study aims to prove whether normal skin fibroblast that is disrupted with mitomycin-C exposure can be repaired with the application of PRF, which contains many growth factors needed for wound healing.

## 2 Material and methods

This study is an in vitro study with quasi-experimental design. This study was done Medical Technology Laboratory, Dermatovenereology Department, Radiopoetro Building 3rd Floor, Faculty of Medicine, Universitas Gadjah Mada, Yogyakarta. The ethical clearance of this study had been approved by the Institutional Review Board of Faculty of Medicine, Public Health and Nursing, Universitas Gadjah Mada (KE/FK/1141/EC). The study was registered at the “Research Registry” with unique identification number UIN of 6456. This study has been reported in line with the Strengthening the Reporting of Cohort Studies in Surgery (STROCSS) criteria [8].

### 2.1 Samples

Research samples were cultured normal human skin fibroblasts taken from the skin part and unexposed to direct sunlight, which is preputium. Preputium skin was obtained from six healthy children undergoing circumcision, whose parents have previously given informed consent.

### 2.2 Experimental procedures

The cultures were taken from the 4th passage, cultured in complete Dulbecco's Modified Eagle's Medium (DMEM) (Merck KGaA, Darmstadt, Germany) contains 5% *fetal bovine serum* (FBS) (Gibco, Life Technologies Corporation), 100 μg/ml Penicillin Streptomycin (Penstrep) (Gibco, Life Technologies Corporation), 100 mg/ml ceftriaxone, and 2,5 μg/ml *Amphotericin B-Fungsione* (Gibco, Life Technologies Corporation), with cell amount of 5 × 10^4^ cell/ml. Those cultured fibroblasts were divided into 5 groups, group I was added to culture media, group II-IV were added with Mitomycin-C. Then, each group was given treatment: (1) Added with culture media, (2) added with culture media, (3) added with PRF 100%, (4) added with PRF 50%, (5) added with PRF 25%. The fibroblast proliferation was measured by 3-(4,5-dimethylthiazol-2-yl)-2,5-diphenyltetrazolium bromide (MTT) assay.

### 2.3 Statistical analysis

Friedman test and post-hoc by the Wilcoxon test were used to evaluate the comparison of the mean optical density was analyzed by. The Wilcoxon Signed Rank test was used to understand the difference between each treatment group. Statistical significance was considered if p < 0.05. Data were presented as mean ± standard deviation. All statistical analyses were performed with the Statistical Package for the Social Sciences program version 17.0 (SPSS Inc, Chicago, Illinois, USA).

## 3 Results

This study used 6 preputium samples from 6 healthy children who underwent circumcision. The obtained preputium were made into fourth passage fibroblasts cultured according to the procedure. Then cell viability test was done to understand the correlation between optical densities acquired with the viable cells amount contained in the wells. The results were as follows:

From those results, curve linear equations for each cell were made to obtain the cell amount formula that can acquire from the optical density value of an examination (Table 1).

Then an experiment was conducted with 90 wells of fibroblast cells (each contained 5000 cells/well) from 6 different subjects as the samples. Fibroblast proliferation was calculated in 5 treatment groups with optical density unit (Table 2), then converted using the linear curve formula and obtained cell amount in each group as follows:

**Table 1:** Correlation test results of optical density with viable fibroblast cells amount.

OD	10.000 cells	5.000 cells	2.500 cells	1.500 cells	1000 cells	0 cell/DMEM
Cell 1	0.649± 0.04564	0.395± 0.034044	0.272± 0.023896	0.205± 0.007371	0.184± 0.014107	0.038± 0.008021
Cell 2	0.592 ± 0.016623	0.404± 0.019157	0.195± 0.009452	0.147± 0.003055	0.142± 0.007767	0.038± 0.008021
Cell 3	0.534± 0.023643	0.389± 0.00755	0.225± 0.022008	0.156± 0.004359	0.130 ± 0.001732	0.038± 0.000577
Cell 4	0.655± 0.017616	0.574± 0.002646	0.411± 0.021127	0.305± 0.007638	0.291± 0.007506	0.041 ± 0.000577
Cell 5	0.507± 0.014526	0.464± 0.023116	0.320± 0.012702	0.279± 0.009644	0.260 ± 0.006245	0.041 ± 0.000577
Cell 6	0.461± 0.017616	0.328± 0.024	0.219± 0.023072	0.160± 0.005033	0.125± 0.003055	0.038± 0.000577

OD: Optical density; DMEM: Dulbecco's Modified Eagle's Medium.

**Table 2:** Optical density measurement of viable fibroblast cells.

Cell 1	10,000 cells	5000 cells	2500 cells	1500 cells	1000 cells	DMEM
	0.598	0.388	0.277	0.208	0.182	0.046
	0.686	0.365	0.293	0.211	0.199	0.03
	0.663	0.432	0.246	0.197	0.171	0.039
Cell 2	10,000 cells	5000 cells	2500 cells	1500 cells	1000 cells	DMEM
	0.574	0.382	0.185	0.15	0.151	0.046
	0.594	0.417	0.203	0.148	0.14	0.03
	0.607	0.413	0.199	0.144	0.136	0.039
Cell 3	10,000 cells	5000 cells	2500 cells	1500 cells	1000 cells	DMEM
	0.531	0.381	0.247	0.159	0.129	0.038
	0.512	0.39	0.226	0.151	0.129	0.038
	0.559	0.396	0.203	0.158	0.132	0.039
Cell 4	10,000 cells	5000 cells	2500 cells	1500 cells	1000 cells	DMEM
	0.67	0.575	0.431	0.307	0.3	0.041
	0.661	0.576	0.414	0.312	0.287	0.04
	0.636	0.571	0.389	0.297	0.287	0.041
Cell 5	10,000 cells	5000 cells	2500 cells	1500 cells	1000 cells	DMEM
	0.506	0.438	0.313	0.268	0.255	0.041
	0.493	0.479	0.313	0.283	0.267	0.04
	0.522	0.477	0.335	0.286	0.258	0.041
Cell 6	10,000 cells	5000 cells	2500 cells	1500 cells	1000 cells	DMEM
	0.472	0.352	0.21	0.161	0.122	0.038
	0.471	0.328	0.246	0.165	0.128	0.038
	0.441	0.304	0.203	0.155	0.126	0.039

From the results, the first group with fibroblast group starved for 24 h then re-cultured with FBS 10% culture medium showed 11.366,56 ± 4.073,32 viable cells. The second group, with Mitomycin-C exposure post-starvation, showed a decrease of 5.690,41 ± 2.834,22 cells. In the group with starvation, exposure of Mitomycin-C and then given PRF with dosage 100%, 50%, and 25% showed an increase compared to the treatment group with Mitomycin-C with 7.909,80 ± 3.392,19; 15.347,91 ± 8.413,02 and 13.449,56 ± 7.523,83 cells respectively.

An evaluation of data distribution normality was done with the Kolmogorov-Smirnov test for data with more than 50 samples, with results in p ≥ 0.05 for medium + FBS 10% group, 100% PRG, 50% PRF and 25% group. P < 0.05 was obtained from the treatment group with Mitomycin-C.

Non-parametrical statistical analysis with the Friedman test was done to the study data to see the comparison of fibroblast proliferation among the five groups. A statistically significant difference was obtained among each group fibroblast proliferation with p = 0.000.

The statistical test was continued with the Wilcoxon Signed Rank test to understand the difference between each treatment groups. Medium + FBS 10% group showed significant difference compared to the treatment group with Mitomycin-C with p = 0.000 and PRF 100% group with p = 0.008. But no significant difference was shown in the 50% PRF group (p = 0.064) and 25% PRF (p = 0.372).

The treatment group with Mitomycin-C reached a significant level compared to 100%, 50%, 25% PRF group with p = 0.022, p = 0.000, and p = 0.000 respectively.

Between PRF treatment groups, the average value of 50% PRF was the highest followed by 25% PRF and 100% PRF. A significant difference was found between 100% PRF compared to 50% PRF group (p = 0.000) and 25% PRF (p = 0.000). Significant difference was also shown in 50% PRF group compared with 25% PRF group with p = 0.012.

## 4 Discussion

**Fig. 1:**
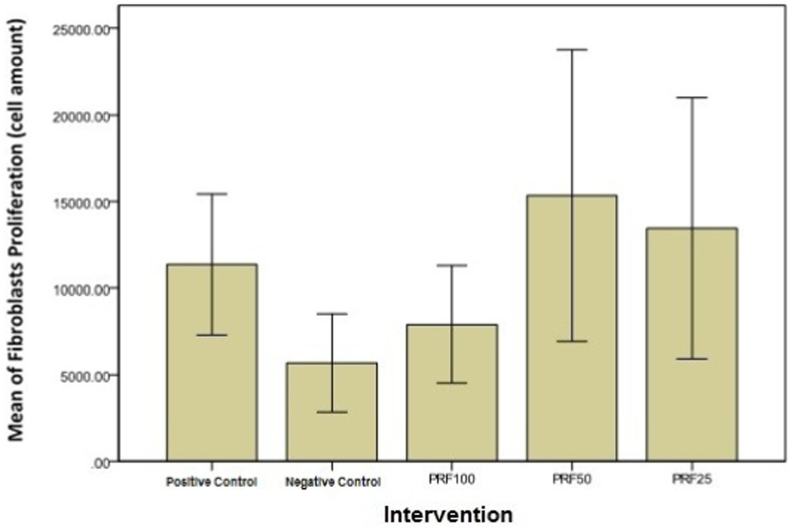
Graph of fibroblast proliferation comparison results among 5 groups. (PRF 100: group with Mitomycin-C added with 100% concentration PRF; PRF 50: group with Mitomycin-C added with 50% concentration PRF; PRF 25: group with Mitomycin-C added with 25% concentration PRF).

We are able to show that this study had significant results in the decrease of fibroblast proliferation ability (Fig. 1). Administration of PRF with different dosages (100%, 50%, and 25%) in this study showed a significant increase of cell amounts compared to the Mitomycin-C treatment group. However, if compared among three PRF treatment groups, the group with 50% concentration of PRF showed the highest increase of cell amount compared with 25% and 100% PRF concentration respectively. This result can be explained that fibroblast and growth factors need certain media and nutrition to live and grow. PRF dilution in this study was made to add DMEM *2x* high glucose and FBS 1% médium with appropriate comparison to obtain 50% and 25% PRF, while those compounds were not added to 100% PRF. Our study is in accordance with previous study by Wang [7] that also showed that PRF significantly increase the cell proliferation when compared to PRP. This might be due to the effect of PRF that contained higher level of growth factor [7].

In this study, PRF with 50% concentration has the highest ability to induce proliferation compared with the other groups (Table 3), although the range was quite wide. This result is probably because of the appropriate combination of growth factor amount with the médium used for the dilution. Physiologically, the real wound is an environment which provides medium and nutrition for the growth of fibroblast and growth factors. Those findings can be a consideration for further in vivo study to see the clinical treatment difference of both chronic and acute wounds with different PRF concentrations.

**Table 3:** Average of cell amount in each treatment groups.

Treatment	Cell Proliferation
Medium + FBS 10%	11.366,56 ± 4.073,32
Mitomycin-C	5.690,41 ± 2.834,22
Mitomycin-C + PRF 100%	7.909,80 ± 3.392,19
Mitomycin-C + PRF 50%	15.347,91 ± 8.413,02
Mitomycin-C + PRF 25%	13.449,56 ± 7.523,83

Our study is not without limitation. First, the PRF used was not autolog with fibroblast (skin for fibroblast culture and vein blood for PRF production were not from the same donor). Many factors can affect the outcome in non autolog condition including local conditions, associated conditions, and factors altering immune and inflammatory responses [9]. Second, the presence of physiological difference between subjects. Some factors are known to be correlated with proliferation, including sex hormones, stress, nutrition and immunocompromised conditions [9]. These limitations could probably affect the conducted study process.

## 5 Conclusions

The study results demonstrate that Platelet-Rich Fibrin can improve normal dermal fibroblast proliferation after treatment with mitomycin-C in vitro. The increasing level depends on the PRF concentration.

## Provenance and peer review

Not commissioned, externally peer-reviewed.

## Funding

This research did not receive any specific grant from funding agencies in the public, commercial, or not-for-profit sectors.

## Consent

Written informed consent was obtained from the parents before joining the study. A copy of the written consent is available for review by the Editor-in-Chief of this journal on request.

## Ethical approval

The ethical clearance of this study had been approved by the Institutional Review Board of Faculty of Medicine, Public Health and Nursing, Universitas Gadjah Mada (KE/FK/1141/EC).

## Author contribution

Ishandono Dachlan, Hendy Satrya Kurniawan and Rosadi Seswandhana conceived the study and critically revised the manuscript for important intellectual content. Aditya Wicaksana, Aditya Rifqi Fauzi and Firdian Makrufardi drafted the manuscript. All authors read and approved the final draft. All authors facilitated all project-related tasks.

## Registration of research studies

1. Name of the registry: Research Registry
2. Unique Identifying number or registration ID: researchregistry6456
3. Hyperlink to your specific registration (must be publicly accessible and will be checked): https://www.researchregistry.com/browse-the-registry#home/registrationdetails/6001091852b9f2001b7f31dd/

## Guarantor

Ishandono Dachlan.

## Declaration of competing interest

No potential conflict of interest relevant to this article was reported.

## Abbreviations

DMEM: Dulbecco's Modified Eagle's Medium
PRF: Platelet-rich fibrin
